# Osteoporosis and fracture as risk factors for self-harm and suicide: a systematic review and meta-analysis

**DOI:** 10.3399/BJGP.2023.0035

**Published:** 2023-09-19

**Authors:** Fay M Manning, Faraz Mughal, Hazem Ahmed Saad Mohamed Ismail, Libby M Baines, Carolyn A Chew-Graham, Zoe Paskins, James A Prior

**Affiliations:** School of Medicine, Keele University, Keele, and Department of Medical Imaging, University of Exeter, Exeter, UK.; School of Medicine, Keele University, Keele; Centre for Mental Health and Safety, Division of Psychology and Mental Health, University of Manchester, Manchester; and honorary clinical research fellow, Warwick Medical School, Warwick, UK.; School of Medicine, Royal College of Surgeons, Bahrain.; School of Medicine, Keele University, Keele, UK.; School of Medicine, Keele University, Keele, UK.; Keele University, Keele, and Haywood Academic Rheumatology Centre, Midlands Partnership Foundation Trust, Stoke-on-Trent, UK.; School of Medicine, Keele University, Keele, and Haywood Academic Rheumatology Centre, Midlands Partnership Foundation Trust, Stoke-on-Trent, UK.

**Keywords:** fracture, general practitioners, meta-analysis, osteoporosis, self-harm, suicide

## Abstract

**Background:**

Increase in presentations of self-harm to primary care, a risk factor of suicide, has led to a growing interest in identifying at-risk populations.

**Aim:**

To examine whether osteoporosis or fractures are risk factors for self-harm, suicidal ideation, and suicide.

**Design and setting:**

This was a systematic review of observational studies in adults (>18 years) that had examined the role of osteoporosis and/or fractures in subsequent self-harm, suicidal ideation, and/or suicide.

**Method:**

Six databases were searched from inception to July 2019. Additional citation tracking of eligible studies was undertaken in November 2022. Screening, data extraction, and quality assessment of full-text articles were performed independently by at least two authors. Where possible, meta-analysis was run on comparable risk estimates.

**Results:**

Fifteen studies were included: two examined the outcome of self-harm, three suicidal ideation, and 10 suicide. In approximately half of studies on osteoporosis, the risk of suicidal ideation and suicide remained significant. However, pooling of adjusted odds ratios from three studies indicated no association between osteoporosis and suicide (1.14, 95% confidence interval = 0.88 to 1.49). Nine studies examined the risk of a mixture of fracture types across different outcomes, limiting comparisons. However, all studies examining vertebral fracture (*n* = 3) reported a significant adjusted negative association for self-harm and suicide.

**Conclusion:**

Patients with vertebral fractures, a risk potential factor for suicide, may benefit from clinical case finding for mood disorders with personalised primary care management. However, because of the limited number and quality of studies and mixed findings, further examination of these associations is warranted.

## INTRODUCTION

Rates of suicide in the UK have risen since 2017, with a 9% increase by 2019.^1^ Similarly, there has been an increase in presentations of self-harm to primary care,^2^ with the risk of suicide increasing 50-fold in the year after self-harm compared with the general population.^3^ Such increases have led to a growing interest in identifying at-risk populations. Although a history of mental ill health, notably depressive disorders, is an understood risk factor for self-harm,^4^ pain, and more specifically back pain, are also independent risk factors for self-harm^5^^,^^6^ and suicide, specifically in older adults.^7^

Recent studies have demonstrated increased risk of self-harm in people with physical health conditions such as fibromyalgia and osteoarthritis.^5^^,^^8^ Although pain is not a symptom of osteoporosis, pain frequently occurs because of subsequent fractures,^9^^,^^10^ and with one in three women and one in five men aged >50 years experiencing an osteoporotic fracture in their lifetime, osteoporosis has been suggested as a risk factor for self-harm and suicide.^5^^,^^11^ Furthermore, common outcomes of fractures, including osteoporotic fractures, such as isolation and depression are risk factors for suicide.^12^

Previous research examining the risk of self-harm or suicide in older adults with osteoporosis or fractures has yielded conflicting results,^5^^,^^13^^–^^15^ possibly because of a lack of consensus on diagnosis definitions, population variations, and study limitations. The aim of this study was to examine whether osteoporosis and/or fractures are risk factors for self-harm, suicidal ideation, or suicide.

## METHOD

A systematic review and meta-analysis was conducted. The protocol was registered on PROSPERO (CRD42021272606) and adhered to PRISMA guidance.^16^

### Search methods

The population of interest were adults with osteoporosis and/or fractures. The outcomes of interest were risk estimates of self-harm, suicidal ideations (including suicidal behaviours), and suicide.

Exclusion criteria were:

- studies with a population of <18 years old;
- studies that could not be translated; and
- systematic reviews, case reports, or case series studies.

**Table:** How this fits in

An increased risk of self-harm and suicide has been identified in people with physical health conditions, such as fibromyalgia and osteoporosis. This is the first study, to the authors’ knowledge, to synthesise the evidence on osteoporosis and fractures as risk factors for self-harm, suicidal ideation, and suicide (including suicidal behaviours). Although there was no association between osteoporosis and suicide, vertebral fractures appeared to increase the risk of self-harm and suicide. Patients with vertebral fractures therefore may benefit from clinical case finding for mood disorders with personalised primary care management.

Searches were tailored and conducted in six electronic databases: MEDLINE, PsycArticles, AMED, CINAHL Plus, PsycINFO, and Web of Science from inception to July 2019. Search strategies utilised database subject headings and text word searching in title, abstract, or keywords, combining terms for: osteoporosis and fractures; and self-harm, suicidal ideation (including suicidal behaviours), and suicide (Supplementary Information S1).

In addition, the reference lists of included studies and relevant systematic reviews were checked and key studies citation tracked. To account for the delay in publication because of COVID-19, citation tracking was conducted on all included papers in September 2021 and November 2022 to ensure relevant new studies were identified.

### Study screening and selection

A two-stage screening of articles against predefined eligibility criteria was implemented: first by titles and abstracts; second by full text.

At each stage, screening was conducted by two reviewers independently (the first, third, and senior author) and articles were excluded by consensus. Arising disagreements were resolved through discussion.

### Quality assessment

The Quality in Prognosis Studies (QUIPS) tool was used to assess included articles.^17^ It examines the risk of bias across six domains: study population, study attrition, prognostic factor measurement, outcome measurement, study confounding, and statistical analysis and reporting. All included articles were assessed for their quality independently by two pairs of assessors (the first and senior author or the second and sixth author). Any disagreement on scoring was initially discussed and arbitrated by a different reviewer if required.

### Data extraction

Data were extracted by two authors (the third and fourth authors) on demographic information (age, sex, country of origin, and so on), and size of the study sample, numbers of patients with the condition of interest, study setting (for example, primary care), exposures (for example, osteoporosis or fracture), exposure definition, outcome (for example, self-harm, suicidal ideation, and suicide), and method of risk estimates regarding the association between exposure and outcome.

Findings were tabulated by their exposure category, that is, articles examining either osteoporosis or fracture were compared separately. Within each of these exposure categories, outcomes were subcategorised by either self-harm, suicidal ideation/behaviour, or suicide. Risk estimates, 95% confidence intervals (CIs), and statistical significance were extracted and reported for each study, and where available separately for men and women.

Specifically for fractures, risk estimates were tabulated by location of fracture, where reported. Authors were not contacted if it was not possible to identify certain required information in their publication.

### Meta-analysis

Where enough studies (≥3) examining comparable factors were identified, a random-effects meta-analysis was used to pool reported risk of any of the examined exposures along with their 95% CI. Heterogeneity was assessed by *I*^2^. Analysis was undertaken in Stata (version 14).

### Patient and public involvement

Keele Research User Group consists of a diverse group of people with lived experience of osteoporosis, or of caring for people with osteoporosis.

In a series of meetings to discuss research, public contributors talked about the significant psychological and social sequelae of osteoporotic fractures. However, there was no direct public and patient involvement in the conduct or interpretation of this study.

## RESULTS

**Figure 1.**
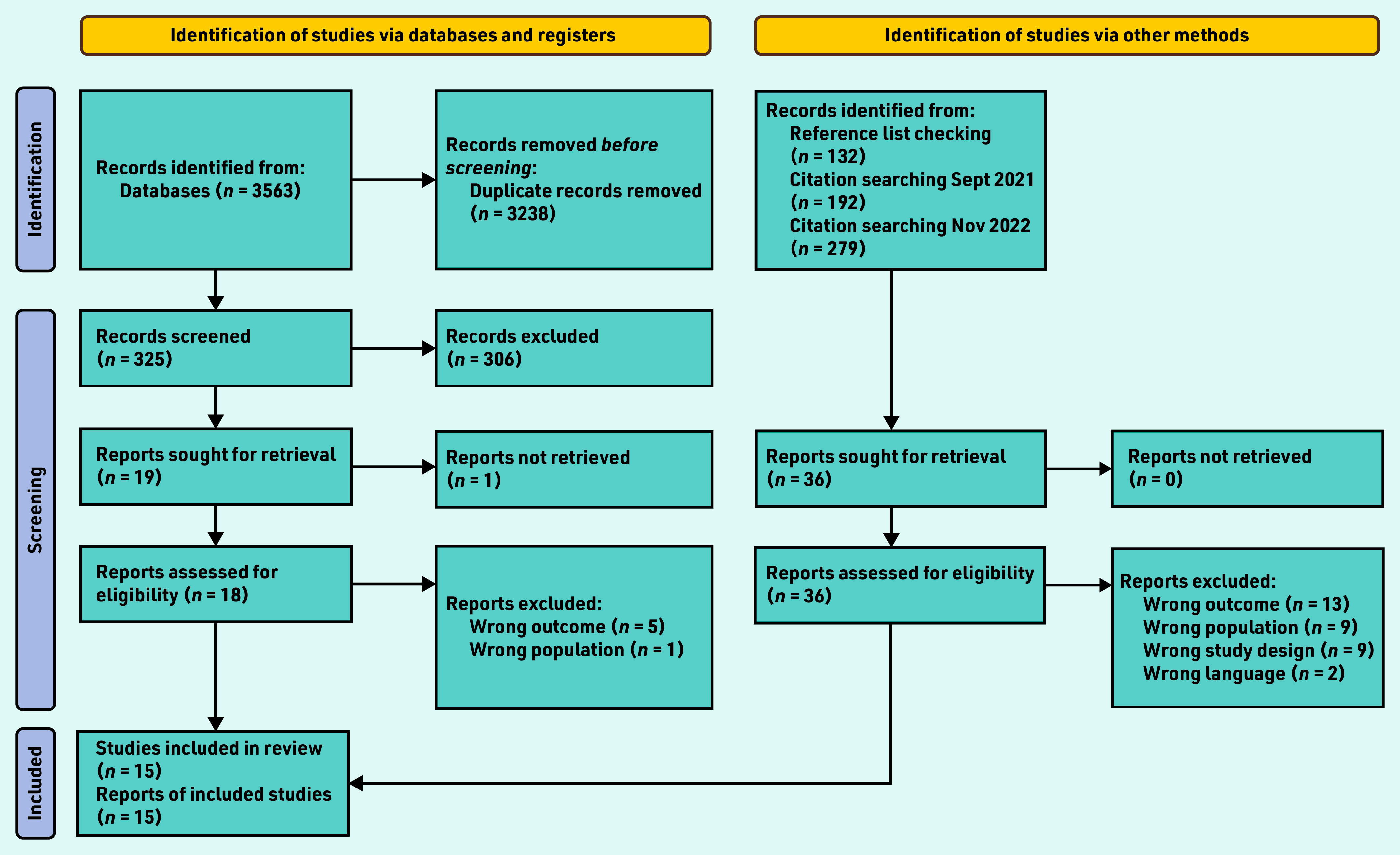
*PRISMA flow diagram for the study.^29^*

Searches identified 325 unique articles, of which 306 were excluded and 18 underwent full review (one article could not be retrieved) (Supplementary Table S1). Citation tracking identified a further three eligible studies resulting in a total of 15 included studies (Figure 1).^11^^,^^13^^–^^15^^,^^18^^–^^28^

The included studies were categorised into two groups by risk factor, osteoporosis, and fracture. Eight studies reported osteoporosis as a risk factor,^11^^,^^13^^,^^14^^,^^18^^–^^22^ 10 studies examined fractures as a risk factor,^13^^–^^15^^,^^20^^,^^23^^–^^28^ and three studies examined both.^13^^,^^14^^,^^20^ Of the studies reporting osteoporosis, three reported suicidal ideation as the outcome,^18^^–^^20^ and five reported suicide as the outcome.^11^^,^^13^^,^^14^^,^^21^^,^^22^ Of the studies examining fractures, two reported the outcome of self-harm,^23^^,^^26^ two of suicidal ideation,^15^^,^^20^ six of suicide,^13^^,^^14^^,^^24^^–^^28^ and one included the outcome of self-harm and suicide.^26^ In the three studies examining both population groups of osteoporosis and fractures, one reported the outcome suicidal ideation^20^ and two reported on the risk of suicide (Table 1).^13^^,^^14^ Most studies included a relatively even sample of male and female participants, but only three stratified risk estimates by sex.^13^^,^^19^^,^^23^ The majority of studies showed moderate to high risk of bias (Supplementary Figure S1).

**Table 1.** Characteristics of included studies

**Study**	**Year**	**Country**	**Study design**	**Setting**	**Sample size (% women)**	**Age, mean (SD) or age groups, years**	**Disease definition/fracture location**
**Osteoporosis and suicidal ideation**							
Chan *et al*^18^	2011	Taiwan	Cross-sectional	General population	3596 (47.4)	73.5 (5.8)	Patient recall of physicians’ diagnosis
Kim *et al*^19^	2016	Korea	Cross-sectional	General population	19 243 (64.8)	19–44, 45–64, 64–74, ≥75^a^	Self-reported diagnosis
Lutz *et al*^20^^,^^b^	2016	Europe	Cross-sectional	General population	38 670 (55.8)	64.88 (10.27)	Patient recall of physicians’ diagnosis

**Osteoporosis and suicide**							
Voaklander *et al*^22^	2008	Canada	Population-based case–control	General population	3601 (28.3)	76.3 (7.08)	Hospital or community record^c^
Webb *et al*^11^	2012	UK	Nested case–control	Primary care	18 333 (33)	17–98^a^	GPRD codes
Erlangsen *et al*^13^^,^ ^b^	2015	Denmark	Register-based cohort	General population	1 849 110 (77.7)	65–79, ≥80^a^	NRP database
Ahmedani *et al*^21^	2017	US	Case–control	Primary and specialist care	270 074 (52.5)	39.4^a^	VDW code
Chang *et al*^14^^,^^b^	2018	Taiwan	Population-based case–control	Primary and secondary health care	173 970 (32)	59.8 (14.3)	Database code (733.0x)

**Fracture and self-harm**							
Erlangsen *et al*^26^^,^ ^d^	2021	Australia	Cohort	General population	266 324 (53.6)	62.7 (11.2)	Bone fracture in past 5 years
Prior *et al*^23^	2021	UK	Respective cohort	Primary care	32 586 (70.1)	74.81^a^	Vertebrae

**Fracture and suicidal behaviour/ideation**							
Lutz *et al*^20^^,^ ^b^	2016	Europe	Cross-sectional	General population	38 670 (55.8)	64.88 (10.27)	Hip/femoral, other
Tsai *et al*^15^	2019	Taiwan	Retrospective cohort	Inpatient/outpatient care	165 608 (43.9)	<35, >65^a^	Trunk, lower limbs, and multiple locations

**Fracture and suicide**							
Turvey *et al*^25^	2002	US	Nested case–control	Retirement community	420 (4.76)	78.6 (76–90)^a^	Hip
Erlangsen *et al*^13^^,^ ^b^	2015	Denmark	Register-based cohort	General population	1 849 110 (77.7)	65–79, ≥80^a^	Spinal, hip, leg, foot, shoulder/arm, and hand
Chang *et al*^14^^,^ ^b^	2018	Taiwan	Population-based case–control	Primary and secondary health care	173 970 (32)	59.8 (14.3)	Pathological, spine, clavicle, humerus, forearm, wrist, radius/ulna/hand, femoral neck/shaft, patella/tibia/fibula, ankle/foot, and pelvis
Jang *et al*^24^	2020	South Korea	Nationwide cohort	Secondary care	34 431 (73.81)	75.0 (6.8)	Hip
Erlangsen *et al*^26^^,^ ^d^	2021	Australia	Cohort	General population	266 324 (53.6)	62.7 (11.2)	Bone fracture
Jang *et al*^28^	2021	South Korea	Nested case–control	NHIS-Senior cohort	18 420 (66.8)	65–>85	Hip, spine, radius, and humerus
Jang *et al*^27^	2022	South Korea	Nested case–control	NHIS-Senior cohort	5198 (65.56)	65–>85	Pelvic fracture

a *No mean and/or SD age of participants provided.*

b *Papers reporting on both osteoporosis and fractures.*

c *Hospital classified as a hospital admission record showing osteoporosis as a primary or contributing diagnosis; community classified as at least two episodes of care recorded for osteoporosis in the physician claim file.*

d *Papers reporting on both self-harm and suicide. GPRD = General Practice Research Database. NHIS = National Health Insurance Service. NRP = National Registry of Patients. SD = standard deviation. VDW = virtual data warehouse.*

### Characteristics of osteoporosis studies

Studies were conducted in a range of countries, predominantly Europe^11^^,^^13^^,^^20^ and East Asia.^14^^,^^18^^,^^19^ Study settings included primary care,^11^ primary and secondary/specialist care,^14^^,^^21^ and general populations.^13^^,^^18^^,^^19^^,^^20^^,^^22^ Four studies did not report average age and/or standard deviation (SD),^11^^,^^13^^,^^19^^,^^21^ with two only reporting the age ranges used to group participants.^13^^,^^19^ Three studies included participants <45 years of age^11^^,^^19^^,^^21^ and four included participants >80 years of age.^11^^,^^13^^,^^19^^,^^22^ Osteoporosis was predominantly identified utilising disease codes in a range of health record databases (Supplementary Table S2). In terms of outcomes, suicidal ideation was recorded via patient self-report^14^^,^^19^^,^^20^ and papers reporting numbers of suicide used healthcare records.^11^^,^^13^^,^^14^^,^^21^^,^^22^

### Osteoporosis as a risk factor for suicidal ideation

Two of the three studies reported significant crude estimates (crude odds ratios [ORs]), ranging from 1.90 (95% CI = 1.38 to 2.62) to 2.13 (95% CI = 1.34 to 3.39) (Table 2).^18^^,^^19^

**Table 2.** Reported crude and adjusted risk estimates for osteoporosis and fractures as a risk factor for self- harm, suicidal ideation/behaviour and suicide

**Study**	**Type of risk estimate**	**Crude**			**Adjusted**		
		**Risk estimate**	**95% CI**	***P*-value**	**Risk estimate**	**95% CI**	***P*-value**
**Osteoporosis and suicide ideation/behaviour**							
Chan *et al* (2011)^18^	OR	1.9	1.38 to 2.62	**Sig**	—	—	—
Kim *et al* (2016)^19^ (men)	OR	2.13	1.34 to 3.39	**<0.05**	2.07	1.19 to 3.59	**<0.05**
Kim *et al* (2016)^19^ (women)	OR	0.86	0.68 to 1.07	N/S	1.07	0.81 to 1.42	N/S
Lutz *et al* (2016)^20^^,^ ^a^	OR	—	—	—	1.19	1.04 to 1.36	**<0.01**
**Osteoporosis and suicide**							
Voaklander *et al* (2008)^22^ (physician coded)	OR	1.69	0.62 to 4.66	N/S	—	—	—
Voaklander *et al* (2008)^22^ (hospital coded)	OR	3.12	1.56 to 6.21	**Sig**	—	—	—
Webb *et al* (2012)^11^	OR	2.33	1.46 to 3.72	**<0.05**	1.62	0.99 to 2.63	N/S
Erlangsen *et al* (2015)^13^ (men)	RR	1.57	1.04 to 2.37	**<0.05**	1.67	1.1 to 2.51	**<0.05**
Erlangsen *et al* (2015)^13^ (women)	RR	1.56	1.25 to 1.95	**<0.001**	1.88	1.5 to 2.35	**<0.001**
Ahmedani *et al* (2017)^21^	OR	1.22	0.92 to 1.63	0.171	1.21	0.9 to 1.62	0.216
Chang *et al* (2018)^14^	OR	1.28	1.23 to 1.33	**<0.0001**	0.97	0.93 to 1.02	0.2582
**Fracture and self-harm**							
Erlangsen *et al* (2021)^26^^,^ ^b^	IR	1.47	—	—	1.38	1.12 to 1.71	**<0.05**
**Fracture and suicidal ideation/behaviour**							
Lutz *et al* (2016)^20^^,^ a^,^ ^c^	OR	—	—	—	1.3	1.1 to 1.54	**<0.01**
Tsai *et al* (2019)^15^	HR	2.37	1.93 to 2.91	**<0.0001**	2.21	1.80 to 2.71	**<0.0001**
**Any fractures and suicide**							
Turvey *et al* (2002)^25^^,^ ^e^	OR	3.39	1.16 to 9.4	**0.02**	—	—	—
Chang *et al* (2018)^14^^,^ ^d^	OR	1.74	1.52 to 1.98	**<0.0001**	1.49	1.28 to 1.73	**<0.0001**
Erlangsen *et al* (2021)^26^^,^ ^b^	IR	1.16	—	—	1.16	0.76 to 1.80	N/S

a *Adjusted odds ratio (Model 1).*

b *Fracture in past 5 years.*

c *Other fracture.*

d *Pathological fracture.*

e *Fracture* >*50 years. CI = confidence interval. HR = hazard ratio. IR = incidence ratio. N/S = outcome was reported as not statistically significant, but no* P*-value was provided. OR = odds ratio. RR = risk ratio*. *Sig = outcome was reported as statistically significant, but no* P*-value was provided.*

Only one of these reported adjusted ORs and they remained significant (for men only 2.07, 95% CI = 1.19 to 3.59, *P*<0.05).^19^ The third study did not report a crude ratio; however, they reported a significant adjusted estimate (1.19, 95% CI = 1.04 to 1.36).^20^

The Kim *et al* study stratified risk by sex,^19^ reporting crude and adjusted risk estimates for suicidal ideation in men and women; however, only risk estimates for males were significant in crude and adjusted ratios (adjusted OR 2.07, 95% CI = 1.19 to 3.59).

### Osteoporosis as a risk factor for suicide

Five of the seven studies relating to osteoporosis as a risk factor for suicide found significant crude results,^11^^,^^13^^,^^14^^,^^21^^,^^22^ with estimates ranging from a crude OR 1.28 (95% CI = 1.23 to 1.33)^14^ to 3.12 (95% CI = 1.56–6.21).^22^ Four studies also reported adjusted RRs, with two retaining significance (males 1.67, 95% CI = 1.1 to 2.51 and females 1.88, 95% CI = 1.5 to 2.35).^13^

**Figure 2.**
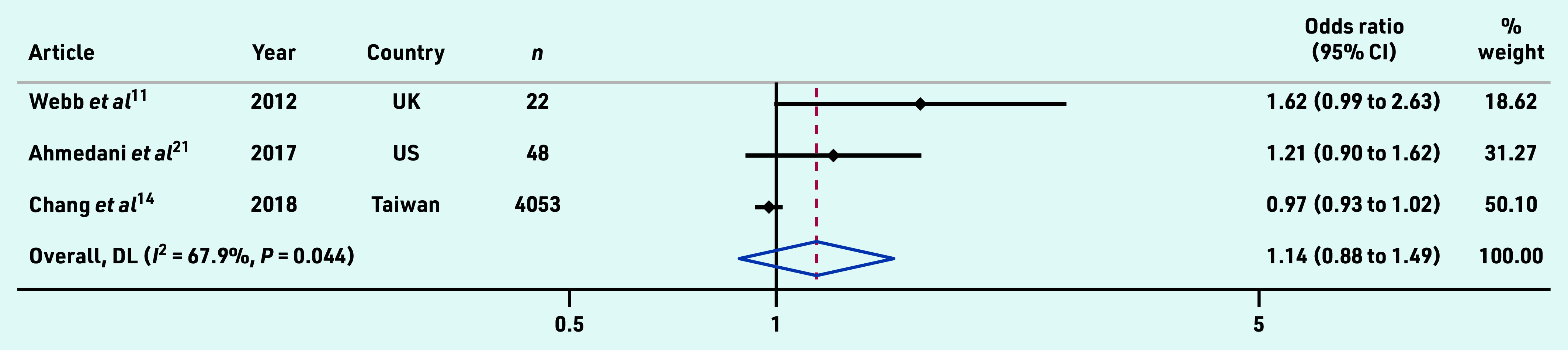
*Pooled adjusted odds ratio estimates from studies examining the association between osteoporosis and suicide. CI = confidence interval. DL = DerSimonian-Laird.*

Adjusted OR data from a total of 4123 primary and secondary care patients across three studies was pooled and found no association between osteoporosis and subsequent suicide (1.14, 95% CI = 0.88 to 1.49) (Figure 2).

### Characteristics of fracture studies

Ten studies, mostly conducted in East Asia^14^^,^^15^^,^^24^^,^^27^^,^^28^ and Europe^13^^,^^20^^,^^23^ examined fracture as a risk factor. Study settings included primary care,^23^ a retirement community,^25^ secondary care,^24^ across primary and secondary care,^14^ and the general population (Table 1).^13^^,^^20^^,^^26^ Most studies included a balanced sample of men and women, or a higher percentage of females (as expected in osteoporotic studies). Four studies reported mean ages ranging from 59.8 (SD 14.3) to 75.0 (SD 6.8) years.^14^^,^^20^^,^^24^^,^^26^ Five studies included participants aged >80 years,^13^^,^^24^^,^^25^^,^^27^^,^^28^ and one included participants below <35 years (Table 1).^15^

Fractures were defined inconsistently between studies. Five of the 10 studies (examining the outcomes self-harm,^13^ suicidal ideation,^15^^,^^20^ and suicide^14^^,^^25^) reported a grouped fracture risk estimate; however, each utilised a different grouping definition: ‘all fractures’, ‘pathological fractures’, ‘fractures >50 years’, ‘other fractures’, and ‘any fracture in last 5 years’ (Table 1 and expanded in Supplementary Table S2). Chang *et al* reported the greatest detail with 11 bodily regions,^14^ whereas Tsai *et al* grouped fractures into just two groups of ‘trunk’ and ‘lower limb’^15^ (Supplementary Table S2). Hip and vertebral fractures were the most consistently reported fracture sites, reported in five and four studies, respectively.^13^^,^^14^^,^^20^^,^^23^^,^^25^^,^^28^ However, these studies utilised a range of risk estimate types (Table 3).

**Table 3.** Crude and adjusted risk estimates of fracture and suicide by fracture location

**Body location**	**Chang *et al* (2018)^14^ OR (95% CI)**	**Erlangsen *et al*^13^ (2015), men RR (95% CI)**	**Erlangsen *et al*^13^ (2015), women RR (95% CI)**	**Jang *et al*^24^ (2020) HR (95% CI)**	**Jang *et al*^28^ (2021) OR (95% CI)**	**Jang *et al*^27^ (2022) OR (95% CI)**	**Turvey *et al*^25^ (2002) OR (95% CI)**
**Spine/vertebrae**							
Crude	**1.94 (1.83 to 2.05)**	**1.51 (1.23 to 1.85)**	**2.06 (1.68 to 2.53)**	—	**1.62 (1.25 to 2.10)**	—	—
Adjusted	**1.53 (1.43 to 1.64)**	**1.51 (1.23 to 1.85)**	**2.20 (1.79 to 2.7)**	—	**1.40 (1.07 to 1.82)**	—	—

**Shoulder/humerus**							
Crude	**1.64 (1.47 to 1.83)**	**1.25 (1.02 to 1.53)**	0.91 (0.78 to 1.07)	—	1.15 (0.48 to 2.80)	—	—
Adjusted	**1.33 (1.17 to 1.51)**	**1.36 (1.12 to 1.67)**	—	—	1.04 (0.42 to 2.59)	—	—

**Forearm**							
Crude	**1.48 (1.38 to 1.60)**	—	—	—	—	—	—
Adjusted	**1.25 (1.15 to 1.36)**	—	—	—	—	—	—

**Wrist**							
Crude	1.18 (0.93 to 1.49)	—	—	—	—	—	—
Adjusted	0.99 (0.75 to 1.31)	—	—	—	—	—	—

**Radius/ulna/hand**							
Crude	**1.39 (1.31 to 1.47)**	1.01 (0.79 to 1.3)	1.04 (0.82 to 1.33)	—	1.02 (0.76 to 1.37)	—	—
Adjusted	**1.15 (1.08 to 1.23)**	—	—	—	0.95 (0.7 to 1.29)	—	—

**Hip/femoral neck**							
Crude	**1.81 (1.62 to 1.95)**	**1.49 (1.25 to 1.78)**	1.35 (1.16 to 1.58)	—	1.31 (0.96 to 1.79)	—	1.45 (0.03 to 10.40)
Adjusted	**1.40 (1.23 to 1.51)**	**1.28 (1.07 to 1.53)**	**1.40 (1.19 to 1.65)**	—	1.21 (0.87 to 1.67)	—	—

**Femoral shaft**							
Crude	**1.93 (1.74 to 2.08)**	—	—	**2.97 (1.32 to 6.69)**	—	—	—

**Pelvis**							
Crude	**2.74 (2.33 to 3.22)**	—	—	—	—	**1.40 (1.07 to 0.82)**	—
Adjusted	**2.04 (1.68 to 2.47)**	—	—	—	—	1.55 (0.95 to 2.54)	—

**Clavicle**							
Crude	**1.83 (1.66 to 2.01)**	—	—	—	—	—	—
Adjusted	**1.42 (1.26 to 1.59)**	—	—	—	—	—	—

**Patella/tibia/fibula**							
Crude	**1.71 (1.59 to 1.84)**	—	—	—	—	—	—
Adjusted	**1.40 (1.29 to 1.53)**	—	—	—	—	—	—

**Leg/lower limb**							
Crude	—	1.20 (0.99 to 1.46)	1.09 (0.88 to 1.35)	—	—	—	—
Adjusted	—	**1.29 (1.06 to 1.57)**	—	—	—	—	—

**Ankle/foot**							
Crude	**1.45 (1.35 to 1.56)**	**1.12 (0.81 to 1.55)**	1.09 (0.74 to 1.61)	—	—	—	—
Adjusted	**1.19 (1.09 to 1.29)**	—	—	—	—	—	—

a *Bodily fracture location groupings primarily based on paper reporting. Significant values denoted in bold. CI = confidence interval. HR = hazard ratio. OR = odds ratio. RR = risk ratio*.

### Fracture as a risk factor for self-harm

Erlangsen *et al* (2021)^26^ investigated ‘any fractures in the prior 5 years’ as a risk factor for self-harm, whereas Prior *et al* examined the relationship between vertebral fractures and self-harm categorised by sex.^23^ Crude and adjusted hazard ratios (HRs) were significant in both men and women. Erlangsen *et al* (2021) found a significant adjusted incidence ratio (IR) of 1.38 (95% CI = 1.12 to 1.71).^26^ Prior *et al* found that men and women with vertebral fractures were almost four and two times more at risk of self-harm than those without such fractures (adjusted HR of 3.90, 95% CI = 1.80 to 8.50 and 1.90, 95% CI =1.10 to 3.20, respectively) (Supplementary Table S3).^23^

### Fracture as a risk factor for suicidal ideation/behaviour

Tsai *et al* reported significant crude (2.37, 95% CI = 1.93 to 2.91) and adjusted HR (2.21, 95% CI = 1.80 to 2.71) estimates for fractures as a risk factor for suicidal ideation (Table 2).^15^ However, when these data were dichotomised by fracture location (trunk or leg/lower limb), no adjusted associations were retained. Lutz *et al* reported a significant adjusted association between experiencing any fractures (defined as anything other than hip/femoral neck) and suicidal ideation (OR 1.3, 95% CI =1.1 to 1.54) (Table 2).^20^

### Fracture as a risk factor for suicide

Five studies examined fractures as a risk factor for suicide. All of these examined one or more specific fracture locations, with three examining the role of ‘grouped’ fractures. Of the grouped fractures, Chang *et al* and Turvey *et al* reported significant crude associations (‘pathological fracture’: crude OR 1.74, 95% CI = 1.52 to 1.98),^14^ ‘fractures in those over 50 years of age’ (crude OR 3.39, 95% CI = 1.16 to 9.4),^25^ but only Chang adjusted for confounders, finding significance to be retained (adjusted OR 1.49, 95% CI = 1.28 to 1.73) Erlangsen *et al* (2021)^26^ examined the adjusted association between self-reported ‘fractures in last 5 years’ and suicide but found no significant association (Table 2).

Three studies examining the role of vertebral fractures on suicide found significant associations in all, with risk estimates ranging from OR 1.40 (95% CI = 1.07 to 1.82) by Jang *et al* to RR 2.20 (95% CI = 1.79 to 2.70) by Erlangsen *et al* (in women).^14^^,^^26^^,^^28^

Five studies examined the association between hip/femoral neck fracture and suicide;^14^^,^^24^^–^^26^^,^^28^ three of these found a significant crude association. Significant associations were retained in two of these after adjustment.^13^^,^^14^ Although several other fracture sites were examined, conflicting or infrequent examination of these means that no consensus on an association with suicide can be drawn (Table 3). Studies examining the risk of self-harm and suicidal ideation in populations with fractures at specific sites were also limited (Supplementary Table S3).

## DISCUSSION

### Summary

This systematic review included all identified studies examining osteoporosis and fracture as risk factors for self-harm, suicidal ideation, and suicide. Although data are limited, particularly for self-harm, this study found that for approximately half of osteoporosis and fracture studies there remained significant risks of suicidal ideation or suicide after adjustment. All three studies that examined the role of vertebral fracture as a risk factor for suicide found a significant association, even after adjustment (although data pooling was not possible). This was also supported by the one study to have examined the role of vertebral fractures on self-harm.^23^

Pooled analysis of three studies found that people with osteoporosis were no more likely to die from suicide than patients without osteoporosis.

### Strengths and limitations

This is the first paper, to the authors’ knowledge, to assemble literature on osteoporosis and fractures as risk factors for self-harm, suicidal ideation, and suicide. Despite only 15 studies being included it was possible to conduct a meta-analysis that provides greater reliability of risk estimates. Another strength is the analysis of fracture as a risk factor by bodily location, which highlighted vertebral fractures as a potentially important risk factor. There are, however, several limitations that need to be recognised. Papers were primarily characterised by population group of either osteoporosis or fractures and, although the poor coding of osteoporosis and fractures in primary care data has previously been noted,^30^ the authors undertook quality assurance to assess such variations (Supplementary Figure S1). A further limitation of this study is that, because of the variety of definitions, populations, and risk estimates used, it was only possible to pool three studies, which varied widely in quality. However, the fact that the meta-analysis included the study by Chang *et al*^14^ that was a very large study, and, that all estimates were adjusted, adds some credibility to these findings and an opportunity for others to build on.

### Comparison with existing literature

Vertebral fractures have significant long-term physical, psychological, and social sequelae. Pain is the likely mechanism driving the association between the presence of a fracture and subsequent self-harm, suicidal ideation, and suicide; especially as osteoporotic fractures have been reported to induce both acute and chronic nociceptive and neuropathic pain.^31^^,^^32^ The presence of fractures has social implications because of the fear of falling or of recurrent fracture,^33^^,^^34^ with osteoporotic fracture increasing the odds of functional decline by 48%.^35^ Further poor osteoporotic fracture outcomes include increased social support requirements and diminished quality of life, including depression and deterioration in perceived health.^36^^,^^37^

Suicide risk is multifactorial, with depression, anxiety, and other mood disorders as major risk factors. As such, a fracture’s impact on patient mental health may in part explain the risk identified related to vertebral fractures.^38^ Furthermore, the effects of fractures last long beyond the time the injury was sustained. Hallberg *et al* reported that, 2 years post-fracture, the social function and mental health components of health-related quality of life were still significantly lower than controls in those with vertebral and hip fractures.^39^ Although this aligns with spinal, and to a lesser extent hip, fractures being significantly associated with suicide in this review, it does not align with the temporal nature of suicide risk post-fracture as reported by Jang *et al*.^27^ They found that risk of suicide was highest in the first 180 days after hip fracture (HR 2.97, 95% CI = 1.32 to 6.69), and then reduced,^24^ with similar patterns in pelvic and spinal fractures.^27^^,^^28^

This lack of association with osteoporosis and suicide may relate to the fact that osteoporosis without fracture is primarily asymptomatic and, although low trauma fracture is an indicator of osteoporosis, not all those who have low bone density have broken a bone. As such, osteoporosis is often referred to as ‘silent’.^9^ Despite this common label, a diagnosis of osteoporosis can cause changes in the perception of one’s self that may result in social isolation, low mood, and anxiety.^34^

### Implications for research and practice

This study indicates that patients diagnosed with vertebral fractures might benefit from case finding for mood disorders, such as depression, a risk factor for suicide, and enquiring about suicide and self-harm.^40^ Osteoporosis is commonly diagnosed and patients seek help for fracture care in primary care, which may therefore be an important setting to identify patients at risk of self-harm and suicide and intervene early. New research would enable the assessment of the feasibility, acceptability, and effectiveness of such approaches. Early intervention studies have shown that physical exercise programmes that focus on physical anxiety can decrease fear of falls in those with osteoporosis, and potentially lead to lower rates of isolation,^41^^,^^42^ which might be a causative factor in self-harm and suicide. Such programmes have been shown to be effectively implemented through primary care.^43^

In conclusion, although studies were typically too diverse for pooling of data, one meta-analysis of three studies showed no association between osteoporosis and suicide. Across several individual studies, vertebral fractures were shown to be potential risk factors for suicide. This review demonstrates the potential importance of teasing apart the role of osteoporosis and fracture in research, and provides strong justification for further research around vertebral fracture in this area. Primary care clinicians could implement case finding for mood disorders and suicide risk assessment in patients with vertebral fractures and undertake subsequent management.
